# High-Quality Genome Sequence of Cystobasidium tubakii JCM 31526^T^, Isolated from East Ongul Island, Antarctica

**DOI:** 10.1128/mra.00741-22

**Published:** 2022-09-15

**Authors:** Masaharu Tsuji, Jun-ichi Ishihara, Atsushi Toyoda, Hiroki Takahashi

**Affiliations:** a Department of Materials Chemistry, National Institute of Technology, Asahikawa College, Asahikawa, Hokkaido, Japan; b Medical Mycology Research Center, Chiba University, Chiba, Chiba, Japan; c Advanced Genomics Center, National Institute of Genetics, Mishima, Shizuoka, Japan; Vanderbilt University

## Abstract

Cystobasidium tubakii has been reported as a new basidiomycetous yeast species from East Ongul Island, East Antarctica. This species does not require amino acids and vitamins for growth and can grow at subzero temperatures. Here, we report a high-quality genome sequence of Cystobasidium tubakii strain JCM 31526^T^, which was isolated from East Antarctica.

## ANNOUNCEMENT

Cystobasidium tubakii was reported as a new basidiomycetous yeast species from East Ongul Island, East Antarctica (1). JCM3256 is the type strain of C. tubakii. The optimum growth temperature for this strain is 15°C to 17°C, and the maximum growth temperature is 25°C (2). C. tubakii also does not require amino acids and vitamins for growth and can grow at −3°C (2). This study analyzed the whole-genome sequence of C. tubakii JCM 31526^T^ (= 9A-1), which was isolated from East Ongul Island, East Antarctica.

The yeast culture and DNA extraction methods are the same as in our previous report (3). Briefly, yeast cells were cultured in yeast extract-peptone-dextrose (YPD) liquid medium (Difco) at15°C for 5 days. Then, the lyophilized cells were powdered in a mortar and used for DNA extraction. The genomic DNA of C. tubakii was extracted and purified using a NucleoBond AXG100 column with a Nucleo buffer set III (TaKaRa Bio) according to the manufacturer’s protocol. The concentration and purity of the genomic DNA were determined with a NanoDrop spectrophotometer (Thermo Fisher Scientific) and the Qubit double-stranded DNA (dsDNA) broad-range (BR) assay kit (Thermo Fisher Scientific). The genomic DNA was then divided into two parts, one for sequencing with the Pacific Biosciences (PacBio) Sequel system and the other for sequencing with the Illumina HiSeq 2500 system. For the PacBio Sequel system, a single-molecule real-time (SMRT) sequence library was constructed using a SMRTbell Express template preparation kit v1.0 (PacBio) according to the manufacturer's protocol. The sequencing library was size selected using the BluePippin system (Saga Science, Beverly, MA, USA) with a minimum fragment length cutoff of 20 kbp. Two SMRT cells were run on the PacBio Sequel system with binding kit v1.0, sequencing reagent kit v2.1, and 360-min movies, yielding a total of 1,305,792 polymerase reads. In this study, default parameters were used except where otherwise noted. PacBio reads were assembled using the FALCON assembler program v0.7 (4) and were used to polish the assembly sequence with SMRT Link v4.0.0.190159 (application: resequencing). For the Illumina HiSeq 2500 system, genomic DNA was fragmented to an average size of 600 bp with the M220 DNA shearing system (Covaris Inc., Woburn, MA, USA). A paired-end library was constructed with a TruSeq DNA PCR-free library preparation kit (Illumina) and was size selected on an agarose gel using the Zymoclean large fragment DNA recovery kit (Zymo Research, Irvine, CA, USA). The final paired-end library was sequenced on the Illumina HiSeq 2500 sequencer with a read length of 250 bp. Illumina HiSeq reads were mapped against the assembled PacBio contigs with BWA-MEM, and single-nucleotide polymorphisms (SNPs) and indels were identified and corrected using Pilon v1.22 (5). A summary of the genomic sequence results for C. tubakii JCM 31526 is presented in Table 1.

**TABLE 1:** Summary of genomic data for Cystobasidium tubakii JCM 31526^T^

Characteristic	Finding for JCM 31526^T^ (= 9A-1)
Sampling site	East Ongul Island
No. of scaffolds	5
No. of reads by HiSeq 2500	4,488,976,000
Coverage (×) by HiSeq 2500	204
No. of reads by PacBio Sequel	12,709,046,936
Coverage (×) by PacBio Sequel	589.3
Total length (Mb)	21.5
GC content (%)	50.1
Scaffold *N*_50_ (kb)	5,243
Avg length (kb)	4,308
GenBank accession no.	BROO00000000.1
DRA accession no.	DRA014414

Antarctica is one of the last frontiers of bioprospecting. We think that genomic information on C. tubakii will be useful in the search for novel cold-active enzymes and bioactive substances.

### Data availability.

This whole-genome shotgun project has been deposited in DDBJ/EMBL/GenBank under BioProject accession number PRJDB13797 with GenBank accession number BROO00000000.1. The raw reads are available under DRA accession number DRA014414.
